# Health-related quality of life after ketamine or esketamine treatment for chronic pain: a review of randomized controlled trials

**DOI:** 10.1016/j.bjane.2026.844838

**Published:** 2026-10-07

**Authors:** Alexandre Vieira Figueiredo, Stefania Lacerda Garcia, Isabel Lima Araújo, Liliane Else Falcão Lins Kusterer, Lucas de Castro Quarantini

**Affiliations:** a Universidade Federal da Bahia, Programa de Pós-Graduação em Medicina e Saúde (PPGMS), Salvador, BA, Brazil; b Universidade de Salvador, Faculdade de Medicina, Salvador, BA, Brazil; c Universidade Federal da Bahia, Faculdade de Medicina da Bahia, Salvador, BA, Brazil; d Universidade Federal da Bahia, Programa de Pós-Graduação em Medicina e Saúde (PPGMS), Laboratory of Neuropsychopharmacology (LANP), Salvador, BA, Brazil

*Dear Editor*,

The management of chronic pain has undergone a profound conceptual transformation, evolving from a symptom-oriented approach to a multidimensional biopsychosocial model in which pain is recognized as a distinct disease entity. Despite advances in the analgesic armamentarium, approximately 22% of patients in primary care worldwide continue to experience debilitating pain that significantly impairs physical function, psychological well-being, and social participation.

In this context, growing clinical interest in N-methyl-D-aspartate (NMDA) receptor antagonists ‒ particularly ketamine and its S-enantiomer, esketamine ‒ reflects the pursuit of multimodal analgesia while mitigating the risks associated with long-term opioid use, one of the primary classes of medications still utilized in chronic pain management. However, although the analgesic efficacy of these agents remains widely debated, their specific impact on Health-Related Quality of Life (HRQoL) remains insufficiently characterized and underrepresented in clinical protocols.

To address this gap, we conducted a systematic review registered in PROSPERO (CRD42024556873), focusing on Randomized Controlled Trials (RCTs) that evaluated HRQoL as a primary or secondary outcome. After rigorous screening of 561 studies across PubMed, Embase, PsycNET, and the Cochrane Library through December 20, 2025, only five studies, comprising a total of 512 patients, met the inclusion criteria. In all included studies, HRQoL was assessed as a secondary outcome. The reconstructed extraction spreadsheet is provided as Supplementary Material. Detailed study characteristics are presented in Table 1. Gemini was used for language editing only and that all scientific content and interpretation are the authors’ own.

**Table 1:** Characteristics, methodological quality, and Health-Related Quality-of-Life (HRQoL) outcomes of randomized controlled trials evaluating ketamine and S-Ketamine in chronic pain.

Author/Year	Study design	Pain condition	Intervention	Comparator	Route	Dose/Regimen	Sample size	HRQoL instrument	Assessment time point	Main HRQoL finding	RoB2 assessment
Vranken et al., 2005	Randomized, placebo-controlled trial	Central neuropathic pain	S(+)-ketamine	Placebo	Iontophoretic transdermal	50 or 75 mg/day for 5 days	33	EQ-5D-5L, SF-36, PDI	Baseline and 1 week	75 mg/day improved EQ-5D, PDI and most SF-36 domains versus placebo; no significant benefit with 50 mg/day.	Low risk
McCaffrey et al., 2018	Multicenter randomized, placebo-controlled trial	Refractory chronic cancer pain	Subcutaneous ketamine + standard therapy	Placebo + standard therapy	Subcutaneous infusion	100–500 mg/day for 5 days	185	FACIT-Pal	Baseline and day 5	Ketamine was associated with poorer HRQoL outcomes than placebo (FACIT-Pal change -3.10 vs. +4.53).	Some concerns in measurement of the outcome
Fallon et al., 2018	Randomized, double-blind, placebo-controlled trial	Cancer-related neuropathic pain	Oral ketamine	Placebo	Oral	40–400 mg/day	214	EuroQoL Thermometer	Throughout study period (AUC)	No significant difference in HRQoL between ketamine and placebo (adjusted p = 0.92).	Low risk
Pickering et al., 2020	Randomized crossover placebo-controlled trial	Refractory neuropathic pain	IV ketamine or IV ketamine + magnesium sulfate	Placebo	Intravenous infusion	Ketamine 0.5 mg/kg over 2h	20	SF-36	Baseline and after each 35-day period	No significant HRQoL differences among ketamine, ketamine/magnesium, and placebo periods.	Low risk
Gupta et al., 2020	Randomized comparative trial	Chronic low back pain with intervertebral disc pathology	Epidural ketamine 50 mg + triamcinolone	Epidural ketamine 25 mg + triamcinolone	Epidural	25 or 50 mg ketamine + triamcinolone	60	RODS	Baseline, 2, 4, 8 and 12 weeks	Greater HRQoL improvement in the 50 mg group compared with the 25 mg group (p = 0.024).	Some concerns due desviation from intended intervention

AUC, Area Under the Curve; EQ-5D-5L, EuroQol 5-Dimension Questionnaire; FACIT-Pal, Functional Assessment of Chronic Illness Therapy-Palliative Care; HRQoL, Health-Related Quality of Life; PDI, Pain Disability Index; RODS, Revised Oswestry Disability Score; SF-36, 36-Item Short Form Health Survey; RoB2, Risk of Bias-2.

The risk of bias was assessed using the Cochrane RoB2 tool. Of the five included studies, three were judged to have a low risk of bias and two a moderate risk: one due to deviations from the intended intervention, as ketamine was administered epidurally,^1^ and the other due to outcome measurement concerns, as the population consisted of patients receiving palliative care.^2^

The physiological rationale for ketamine use in chronic pain lies in its ability to modulate central sensitization. Activation of NMDA receptors plays a central role in this process by promoting synaptic plasticity and the maintenance of chronic pain states. By antagonizing these receptors, ketamine and esketamine may attenuate sensitization and increase pain thresholds. In addition, ketamine modulates descending monoaminergic inhibitory pathways and interacts with opioid receptors, suggesting a synergistic mechanism capable of restoring functional capacity through multiple neurochemical pathways.

Given the marked heterogeneity of pain phenotypes, instruments used to measure HRQoL, therapeutic regimens, and routes of administration, a meta-analysis was not performed. The results were synthesized according to the Synthesis Without Meta-analysis (SWiM) recommendations. Of the included studies, two demonstrated improvements in HRQoL after esketamine or ketamine treatment, two showed no difference, and one demonstrated worsening quality of life. None of the articles included or cited in this systematic review have been retracted or are currently subject to expressions of concern.

Vranken et al. reported significant improvements in HRQoL, as measured by the SF-36 and EQ-5D-5L, following iontophoretic transdermal administration of S-ketamine in patients with refractory central pain.^3^ Notably, these improvements occurred in the absence of significant reductions in Visual Analogue Scale (VAS) scores. The effect was observed in the group receiving 75 mg/day for 5 days compared with placebo (p < 0.025). These findings challenge the traditional assumption that improvements in quality of life are linearly dependent on reductions in pain intensity.

Gupta et al. showed significant improvements in HRQoL scores after treatment of low back pain with a single 50 mg dose of epidural ketamine, assessed using the RODS instrument at all follow-up time points, with the most pronounced improvement observed after 12 weeks (p = 0.024).^1^

The route of administration appears to be a critical determinant of therapeutic efficacy. Studies using oral or subcutaneous ketamine have generally yielded less favorable outcomes. Fallon et al. reported no significant improvement in HRQoL with oral ketamine in patients with cancer-related neuropathic pain.^4^ This may be explained by ketamine’s low oral bioavailability (∼16.5%) and extensive first-pass metabolism. Consequently, plasma concentrations may be insufficient to sustain the effects required for meaningful functional improvement.

Similarly, Pickering et al. found no statistically significant difference when comparing intravenous ketamine at a dose of 0.5 mg/kg administered every 35 days with placebo in patients with neuropathic pain. The SF-36 instrument was used to assess HRQoL every 7 days (p = 0.804).^5^

McCaffrey et al. reported a worsening of HRQoL in a study of subcutaneous ketamine administered at progressive doses of 100, 300, and 500 mg over 5 days in patients with cancer pain receiving palliative care. HRQoL was measured using the FACIT-Pal instrument (p < 0.01).^2^ This result was mainly driven by adverse effects such as cognitive impairment, dizziness, and fatigue. In vulnerable populations, the neurocognitive burden may outweigh the analgesic benefit, highlighting the importance of careful patient selection.

An important confounding factor in interpreting these findings is the well-established efficacy of ketamine in the treatment of Major Depressive Disorder (MDD) and Treatment-Resistant Depression (TRD). Given the high prevalence of depression among patients with chronic pain, it is plausible that the observed improvements in HRQoL are mediated, at least in part, by ketamine’s rapid antidepressant effects. By promoting synaptic plasticity and reducing depressive symptoms, ketamine may facilitate behavioral activation and functional recovery independently of its direct analgesic effects. This raises a fundamental question for future research: does ketamine improve quality of life primarily by alleviating pain, or by reducing the psychological burden associated with chronic pain? Its rapid effects on mood may create a “therapeutic window” that enables greater patient engagement in rehabilitation strategies. Safety and long-term outcomes must also be considered.

HRQoL measurement tools differ in their emphasis on physical, emotional, and social domains, which limits comparability and precludes a formal meta-analysis. Additionally, variability in routes of administration, dosing regimens, and follow-up durations prevents the establishment of standardized maintenance protocols.

In conclusion, ketamine and esketamine represent promising therapeutic options for refractory chronic pain; however, their impact on HRQoL is highly dependent on appropriate patient selection, route of administration, and an integrated understanding of their dual analgesic and psychotropic effects. Future studies should prioritize HRQoL as a primary endpoint and employ standardized, multidimensional instruments. Only by moving beyond numerical reductions in pain can we fully assess the clinical value of these NMDA receptor antagonists in restoring patient function and well-being.

## Authors' contributions

Alexandre Vieira Figueiredo: This author contributed to conceptualization, investigation, data collection, methodology, project administration, supervision, and writing-original draft, review & editing. Stefania Lacerda Garcia: This author contributed to conceptualization, investigation, data collection, methodology, project administration, and writing-original draft, review, and editing. Isabel Lima Araújo: This author contributed to conceptualization, investigation, data collection, methodology, project administration, and writing-original draft, review, and editing. Liliane Else Falcão Lins Kusterer: This author contributed to conceptualization, investigation, data collection, methodology, project administration, co-supervision, and writing-original draft, review & editing. Lucas de Castro Quarantini: This author contributed to conceptualization, investigation, data collection, methodology, project administration and guidance, supervision, and writing-original draft, review & editing.

## Conflicts of interest

The authors declare no conflicts of interest.

## Data Availability

No new data were generated or analyzed in this article. Data sharing is not applicable to this article.

## Appendix Supplementary materials

Image, application 1

## Footnotes

Supplementary material associated with this article can be found in the online version at doi:10.1016/j.bjane.2026.844838.
