# Supplementary material for: Health-related quality of life after ketamine or esketamine treatment for chronic pain: a review of randomized controlled trials
Source: Braz J Anesthesiol. 2026 Oct 7;77(1):844838. doi: 10.1016/j.bjane.2026.844838 (PMC13742210; doi:10.1016/j.bjane.2026.844838)
Supplement: Supplementary file 1 [file mmc1.docx]

BJAN-D-26-00402_Supplementary Material

**Supplementary Material and responses to the reviewers' and editor's comments**

**Search strategies**

A systematic literature search was conducted using PubMed/MEDLINE, Embase, PsycNET, and the Cochrane Library to identify relevant publications through December 20, 2025, focusing on quality-of-life measures in chronic pain patients treated with ketamine and its enantiomers.

The search strategy incorporated the following terms: ("Chronic Pain" OR "Widespread Chronic Pain" OR "Chronic Primary Pain" OR "Chronic Secondary Pain" OR "Cancer Pain" OR "Tumor-Related Pain" OR "Oncological Pain" OR "Myofascial Pain Syndrome*" OR "Neuralgia" OR "Neuropathic Pain" OR "Nerve Pain") AND ("Ketamine" OR "Esketamine" OR "S-Ketamine" OR "Spravato" OR "Ketalar" OR "Ketanest") AND ("SF-36" OR "EQ-5D" OR "WHOQOL" OR "Quality of Life" OR "Health-Related Quality of Life" OR "HRQOL").

Gray literature was excluded, as we considered only published, peer-reviewed studies. To exclude animal studies, the Cochrane filter for human studies was applied. Additionally, Correct the supplementary text on filters. keyword and publication-type filters were used to exclude systematic reviews. No conference abstracts or unpublished clinical trials were sought.

**Eligibility criteria**

All Randomized Controlled Trials (RCTs) were eligible for inclusion. Non-randomized study designs ‒ such as prospective or retrospective cohort studies, case-control studies, case series, review articles, letters, meta-analyses, case reports, and commentaries ‒ were excluded.

Studies whose target population consisted of children (under 18 years-old) were excluded, as well as any articles in which ketamine or its enantiomers were used as an analgesic strictly in a prophylactic, perioperative manner. Furthermore, non-English publications, studies available only as abstracts without full-text access, and reports containing overlapping patient data from other studies were excluded.

**Data extraction**

Two researchers (AF, SG) independently conducted the literature search and study selection. In cases of disagreement consensus was achieved through discussion and consultation with a senior researcher (LQ). An examination of the literature was undertaken to verify data accuracy, across the text, tables, figures, and supplementary materials.

Data extraction was similarly carried out by two independent researchers (AF, SG), who collected detailed information regarding publication specifics (lead author, publication year, and study design), patient demographics (total number of patients, group sizes, and age), the use of racemic ketamine or any specific isomer, route of administration, dosing regimen, type of chronic pain, and relevant outcomes. Disagreements were resolved through consensus and consultation with a senior researcher (LQ).

**Study inclusion**

Note on study inclusion: Data regarding HRQoL for McCaffrey et al. (Economic evaluation of the randomized, double-blind, placebo-controlled study of subcutaneous ketamine in the management of chronic cancer pain. Palliat Med. 2019 Jan; 33(1):74-81. doi: 10.1177/0269216318801754) were extracted from its economic evaluation report. This was necessary because the original clinical trial by Hardy et al. (Randomized, double-blind, placebo-controlled study to assess the efficacy and toxicity of subcutaneous ketamine in the management of cancer pain. J Clin Oncol. 2012 Oct 10;30(29):3611-7. doi: 10.1200/JCO.2012.42.1081) evaluated solely the efficacy and toxicity of ketamine in chronic pain management and did not assess HRQoL within the selected sample.

**Figure 1** PRISMA 2020 Flow Diagram. This figure shows the workflow of the screening process. The titles and abstracts of 561 studies were screened, and 32 were deemed potentially eligible for our research. The full text of these studies was examined, and 5 were considered eligible for extraction. From: Page MJ, McKenzie JE, Bossuyt PM, Boutron I, Hoffmann TC, Mulrow CD, et al. The PRISMA 2020 statement: an updated guideline for reporting systematic reviews. BMJ 2021;372:n71. doi: 10.1136/bmj.n71.

**Figure 2** Risk of bias assessment of included studies using RoB2.0.

**Risk of bias assessment**

The assessment of methodological bias in the analyzed studies was conducted using the Cochrane Risk-of-Bias tool for randomized trials (RoB2) framework. Two authors performed this evaluation. In the event of a dispute, a third author was available to make the final decision. The RoB2 tool (attached in the supplementary material) was utilized to examine potential biases in the design, execution, and reporting of the included studies.

The study by Gupta et al. (2020) shows a moderate risk of bias due to deviations from the intended intervention, as the authors utilized epidural ketamine. In the current literature, there is limited evidence regarding the efficacy and safety of ketamine via this route of administration.

The study by McCaffrey et al. (2018) presents a moderate risk of bias due to the measurement of the outcomes. Because the sample comprised patients receiving palliative care, the advanced clinical conditions of these patients may have influenced the results.

**Responses to the editor's and reviewers' requests**

References: The references for the five studies included in this systematic review have been properly inserted into the manuscript.

Table 1: Table 1 has been modified to incorporate all the suggested elements and has been embedded in the manuscript.

SWiM Approach: The Synthesis Without Meta-analysis (SWiM) guidelines have been adopted and integrated into the manuscript.

Study Title: The title of the study has been changed exactly as suggested.

Term “Esketamine” in the Title: The study by Vranken et al. (2005) investigated the S-isomer of ketamine, which justifies the inclusion of “esketamine” in the manuscript's title.

AI Disclosure: We have added the following one-line statement to the manuscript: “The authors used Gemini for language editing only; all scientific content and interpretation are the authors’ own”.

Retraction Check. Request: We kindly ask you to carefully verify whether any of the studies cited in your manuscript have been retracted. Response: We checked all included and cited studies against the Retraction Watch Database, PubMed/MEDLINE, and publishers' websites. We are pleased to confirm that none of the articles included or cited in this systematic review have been retracted or are currently subject to expressions of concern. This search was conducted on March 20, 2026.

Data Spreadsheet: Since the original working extraction spreadsheet was not retained after the completion of the review, we reconstructed the complete data extraction table directly from the source studies. We used the Rayyan platform at this stage of the review. The attached spreadsheet fully reflects the data used in the review and all subsequent analyses. The Excel spreadsheet containing the extracted study data is provided as Supplementary Material.

Editor's Suggestions: All suggestions made by the editor have been successfully addressed and incorporated into the manuscript.

Thank you for your valuable guidance and understanding.
